# Current and Future Treatment Strategies for Rhabdomyosarcoma

**DOI:** 10.3389/fonc.2019.01458

**Published:** 2019-12-20

**Authors:** Celine Chen, Heathcliff Dorado Garcia, Monika Scheer, Anton G. Henssen

**Affiliations:** ^1^Department of Pediatric Oncology/Hematology, Charité-Universitätsmedizin Berlin, Berlin, Germany; ^2^Pediatrics 5, Klinikum Stuttgart, Olgahospital, Stuttgart, Germany; ^3^Berlin Institute of Health, Berlin, Germany; ^4^German Cancer Consortium (DKTK), Partner Site Berlin, German Cancer Research Center (DKFZ), Heidelberg, Germany; ^5^Experimental and Clinical Research Center (ECRC) of the MDC and Charité Berlin, Berlin, Germany

**Keywords:** rhabdomyosacoma, pediatric oncology, soft tissue sarcoma, targeted therapy, childhood cancer

## Abstract

Rhabdomyosarcoma (RMS) is the most common soft tissue sarcoma in children, and can be subcategorized histologically and/or based on PAX-FOXO1 fusion gene status. Over the last four decades, there have been no significant improvements in clinical outcomes for advanced and metastatic RMS patients, underscoring a need for new treatment options for these groups. Despite significant advancements in our understanding of the genomic landscape and underlying biological mechanisms governing RMS that have informed the identification of novel therapeutic targets, development of these therapies in clinical trials has lagged far behind. In this review, we summarize the current frontline multi-modality therapy for RMS according to pediatric protocols, highlight emerging targeted therapies and immunotherapies identified by preclinical studies, and discuss early clinical trial data and the implications they hold for future clinical development.

## Introduction

Rhabdomyosarcoma (RMS) is the most common soft tissue sarcoma in children, comprising 4.5% of all childhood cancer with an annual incidence of 4.5 cases per 1 million children (1, 2). RMS cells resemble skeletal muscle progenitor cells, though they can arise from non-skeletal tissue origins (3). RMS is historically classified based on histopathologic features into distinct clinical subtypes— embryonal RMS (ERMS), alveolar RMS (ARMS), pleomorphic, and spindle cell and sclerosing RMS (ssRMS) (4, 5). ERMS represents the majority of cases and is associated with a favorable prognosis, while ARMS is more clinically aggressive due to a propensity for metastasis and recurrence (6–8). Eighty percentage of ERMS tumors are characterized by a loss of heterozygosity at the 11p15 locus, and they generally represent a more biologically heterogeneous group of tumors compared to ARMS (1). The recently recognized subtype, spindle cell and sclerosing RMS is a rare variant of RMS characterized by recurring fusions of *VGLL2* or *NOCA2* and has a favorable prognosis, so it is treated without the aggressive multimodal regimens used to treat ARMS and ERMS (5). In this review, we focus on the ARMS and ERMS subtypes.

The majority of ARMS tumors harbor a recurrent chromosomal translocation, *t*(2;13)(q35;q14) or *t*(1;13)(p36;q14). The 2;13 and 1;14 translocations encode for a chimeric transcription factor (TF), consisting of the N-terminal DNA binding domain of PAX3 or PAX7 fused to the C-terminal transactivation domain of FOXO1 (9, 10). Of all ARMS patients, approximately 60% express PAX3-FOXO1, 20% express PAX7-FOXO1, 20% are fusion negative (11, 12), and a small subset express rare variants such as PAX3-FOXO4 or PAX3-NOXA1 (12). Patients with the *PAX7-FOXO1* rearrangement have superior overall survival (82%) compared to patients with the *PAX3-FOXO1* rearrangement (61%) (12). Notably, chromosomal amplification was reported in the majority (93%) of *PAX7-FOXO1* cases compared to *PAX3-FOXO1* (9%) (13), raising the question of whether fusion gene amplification is linked to more favorable outcomes. At this point in time, it is unknown whether the PAX7 fusion partner or gene amplification is the main determinant of favorable outcome, but prospective tracking of fusion gene amplification in COG study ARST1431 is expected to clarify if gene amplifications contribute toward the observed difference. The remaining 20% of fusion-negative ARMS tumors present a similar molecular profile and clinical outcome to the ERMS subtype (14–16). The PAX-FOXO1 chimeric protein behaves as a highly active transcription factor to drive aberrant gene expression, encoding for downstream gene targets required for oncogenic transformation. The oncogenic capacity of the PAX-FOXO1 fusion proteins has been well characterized by multiple studies and has been shown to act as a dominant-acting oncogene in driving tumorigenesis in fusion-positive RMS (FP RMS) (4, 17). On the other hand, fusion-negative RMS (FN RMS) is characterized by higher rates of aneuploidy and single-nucleotide variations, with the RAS pathway most commonly activated in the majority of FN tumors (18–20). There is a known link between RMS and cancer predisposition syndromes, such as Li-Frameni syndrome, neurofibromatosis, Beckwith-Wiedemann syndrome, and Costello syndrome (19).

**Figure 1:**
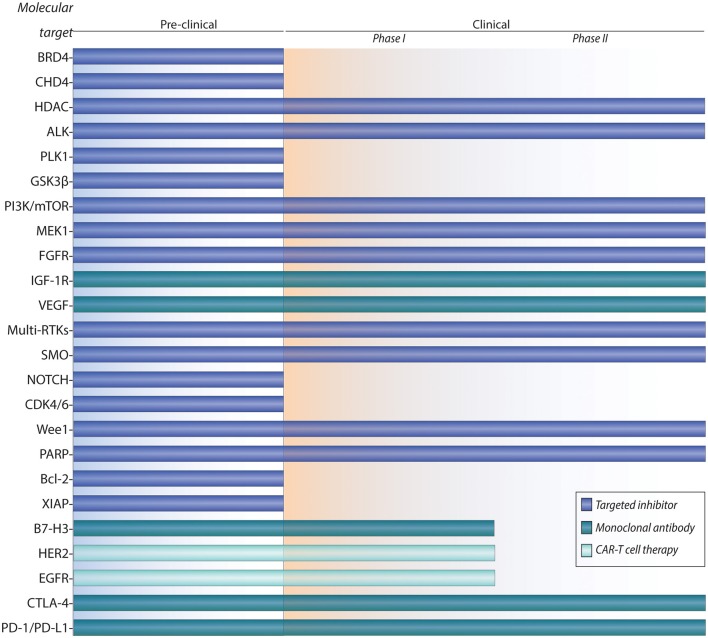
Pipeline of preclinical and clinical development for targeted therapies and immunotherapies of rhabdomyosarcoma.

Over 90% of patients with low-risk localized disease can be cured with multi-modal therapy, but overall survival rates of patients with metastatic or recurrent disease remain dismal at 21% and 30%, respectively (21, 22). Because RMS is a rare disease, cooperative trials in Europe (European pediatric Soft Tissue Sarcoma Study Group (23), Cooperative Weichteilsarkom Studiengruppe der Gesellschaft für pädiatrische Onkologie und Hämatologie (CWS) (21, 23) and North America (Children's Oncology Group) (24) have been crucial for clinical study of this disease. Given that no significant improvements in the survival outcomes of metastatic and recurrent RMS patients in the last 30 years have been reached, there is an unmet need for novel treatment paradigms. Moreover, RMS patients could benefit from molecularly targeted and immunotherapeutic approaches, which could reduce the treatment-associated toxicities caused by current chemotherapy and radiation therapy (RT). Because funding of drug development for a rare childhood cancer such as RMS is limited, preclinical studies have focused on molecularly actionable targets that have been studied in other human cancers, many of which have clinically approved therapies. Here, we review the current frontline therapies, followed by an overview of emerging targeted therapies and immunotherapies in RMS (Figure 1).

## Frontline Therapy

The last five decades of cooperative group trials for RMS have improved the 5-year overall survival of patients with pediatric RMS, which now exceeds 70% (25–28). These improvements follow collaborative group clinical trial efforts, which have enabled improvements in chemotherapeutic dosing regimens, local control, and management of treatment-related toxicities. However, improvements in cure rate have generally been limited to patients with low- and intermediate-risk RMS, while no significant progress has been reached in cure rates for patients with advanced or metastatic RMS. Both European and American cooperative group studies have developed more sophisticated risk stratification systems to include more comprehensive prognostic features [patient age, tumor size and site, lymph node involvement, and/ or metastases and surgical group classification (IRS)] that allow more personalized and effective treatment approaches (29, 30).

The support for inclusion of fusion status for risk stratification in clinical trials has been disputed by conflicting studies. A study by Missiaglia et al. reported that positive fusion status correlated with an inferior clinical outcome, while Stegmaier et al. reported there to be no correlation between fusion status and clinical outcome (31, 32). The inconsistent results from these retrospective studies can be partly explained by methodological biases of convenience sampling, where samples are not truly representative of the whole population but rather chosen based on archival sample availability (33–35). Support for the inclusion of fusion status in future clinical trials comes from retrospective analyses such as one by Selfe et al., which argued that re-assignment based on fusion status could spare a significant number of patients from treatment-associated toxicities caused by unnecessary intensive therapy (36). Prompted by conflicting results from previous studies, in 2019 the COG re-examined the prognostic importance of fusion status and determined it was the second most important prognostic factor, after metastatic status (37). Taken together with additional supporting evidence for the inclusion of fusion status as a significant prognostic marker (31, 38) and evidence that FN ARMS and ERMS are molecularly indistinguishable (16), ARST1431 was the first COG trial to use fusion status instead of histopathological status (39). On the European side, fusion gene status will now be used instead of histological status for risk stratification in EpSSG trials (40). It should be mentioned that currently in Europe and North America, high-risk stratification is assigned based on metastatic status, irrespective of fusion status and histology. Whereas, previous studies focused on fusion status as an important prognostic marker in low- and intermediate-risk RMS (38, 41, 42), a review of high-risk RMS cases found that fusion status does not offer the same level of predictive value for metastatic patients. In cases of metastatic RMS, clinical risk factors remain the major predictors of outcome.

The current frontline treatment for all risk-groups of RMS is a multi-modal approach, comprising chemotherapy, surgical resection, and/or radiation therapy. In North America, the standard chemotherapy backbone includes vincristine, actinomycin D, and cyclophosphamide (VAC) (43, 44) and in Europe, the backbone consists of isofasfamide, vincristine, and actinomycin D (IVA) (45). A randomized trial confirmed there to be no significant difference in patient outcomes between the two treatment combinations, so VAC and IVA have continued to be used in their respective regions (25). Since the VAC/IVA regimen was first established four decades ago, the chemotherapy backbone has remained the same besides changes in duration, dosage, and route of administration. After three decades of controversy regarding the inclusion of doxorubicin in the chemotherapy regimen (43, 46–48), an open-label phase 3 trial (EpSSG RMS 2005) conclusively showed that addition of doxorubicin to the standard IVA backbone did not improve patient outcomes in high-risk rhabdomyosarcoma (49). Because of doxorubicin's known risk of cardiotoxicity (especially in younger patients), there is a lack of justification for its continued inclusion in the chemotherapy regimen.

### Localized RMS

Today, children with low-risk RMS (localized to favorable anatomical sites, grossly resected ERMS) treated with frontline multi-modality therapy have excellent outcomes (90% relapse-free survival). Recent clinical research on low-risk RMS has focused on reducing toxicity of treatment by decreasing dosage and duration of alkylating agent, without compromising the ability to prevent disease recurrence. The alkylating agent, cyclophosphamide used in the VAC chemotherapy regimen is known to cause acute and late effects, including severe myelosuppression, infectious complications, and infertility (50). Aiming to minimize treatment-related toxicities, the COG ARST0331 study showed that shorter duration therapy (which included lower-dose cyclophosphamide) and radiation therapy (RT) did not compromise failure-free survival for the majority of patients (51).

Eradication of gross primary tumor is achieved by a combination of surgery and/or RT, in addition to the standard systemic chemotherapy backbone. RT is included in the frontline treatment for nearly all RMS patients, although long-term toxicity poses a significant concern in younger patients (52). Strategies to reduce radiation-related toxicities include incorporation of intensity-modulated RT (IMRT) or proton bean RT and the use of brachytherapy for specific sites e.g., bladder or vagina, both of which are assumed to reduce late toxicities (skeletal muscle/soft tissue changes, joint stiffness, skeletal growth problems, and secondary malignancy) (53–56). It is worth noting a difference in treatment philosophies between North American and European studies. European studies use overall survival as the study end-point, preferring less aggressive local treatment (omission of RT if possible) to mitigate late toxicities, while tolerating a greater risk of relapsed disease. Conversely, North American studies tend to focus on event-free survival as the study end-point, so treatment strategies favor more aggressive local treatment with radiation therapy (1).

In localized high-risk RMS, the benefit of additional maintenance therapy is currently investigated by the two European groups EpSSG and CWS in two international trials. The EpSSG reported an improved overall survival with cyclophosphamide/vinorelbine in the first preliminary assessment at the end of the recruitment period of EpSSG RMS2005 (2008–2013) (57). The ongoing CWS-2007HR trial is a randomized study of whether the addition of an oral maintenance regimen of O-TIE (etoposide, idarubicin, tofosfamide) can benefit patients with localized high-risk RMS. The EpSSG RMS 2005 randomized phase III trial reported that addition of maintenance chemotherapy improved survival for patients with non-metastatic high-risk RMS (58, 59). Since this study was published in 2019, vinorelbine/cyclophosphamide maintenance chemotherapy has been established as the new standard-of-care for treatment of localized high-risk RMS.

### Metastatic RMS

Survival outcomes for patients with metastatic disease remain dismal (event free survival <20%, excluding patients <10 years old diagnosed with ERMS), and the frontline treatment has not advanced significantly over the last 30 years (22, 29, 60). Today in Europe and the United States, the Oberlin score is used for risk stratification and classification of metastatic RMS, assigning a risk score based on patient age, primary tumor site, number of metastases, histology, and bone marrow involvement (29). Unlike for localized disease, for metastatic RMS multimodal therapy frequently fails due to lack of a proper local therapy to treat metastatic sites such as the bone marrow and lungs. A European trial for metastatic RMS reported that high-dose chemotherapy (HD-CT) did not significantly improve survival outcomes compared to standard chemotherapy, despite increased treatment-associated toxicities (61). This report is consistent with a retrospective analysis of 389 patients, which found no significant improvements in survival after HD-CT with hematopoietic stem cell rescue in the treatment of metastatic rhabdomyosarcoma (62). Results from the COG ARST0431 trial for patients with high-risk RMS found that high-dose chemotherapy (dose-compressed cycles of ifosfamide/etoposide and vincristine/doxorubicin/cyclophosphamide, irinotecan, and radiation) did not produce meaningful benefit for most patients, except for a minority of patients with embryonal histology and limited metastatic disease (restricted to lungs) (63). Notably, the inefficacy of HD-CT has also been documented in trials for other metastatic childhood solid tumors, including Ewing sarcoma and osteosarcoma (64–66). Observations from a trial conducted on pediatric neuroblastoma patients treated with HD-CT and stem cell transplantation rescue found there were long-term health consequences (hearing loss, gonadal insufficiency) associated with treatment (67). An important recent study by Merker et al. reported that haploidentical allogeneic hematopoietic stem cell transplantation (HSCT) has inferior outcomes compared to standard maintenance therapies, such as O-TIE (68). Here, the authors argue against the use of allogeneic HSCT, which has more severe side effects than standard maintenance therapy. Taken together, HD-CT and allogeneic HSCT should be discontinued because they failed to achieve curative effects in metastatic RMS, and similar outcomes can be achieved with the less toxic maintenance therapies.

Ongoing European studies investigating the role of maintenance therapy are based off a report by Klingebiel et al. for the HD CWS-96 study. The HD CWS-96 trial was a non-randomized trial comparing the efficacy of high dose therapy (HDT) vs. oral maintenance therapy (OMT) in patients with stage IV soft tissue sarcoma (69). While high dose therapy failed to improve survival, oral maintenance therapy was a promising alternative, since the oral administration can provide long lasting exposure to chemotherapy without increasing toxic side effects. As such, an OMT regimen of O-TIE is the current standard of care within the CWS, while an OMT combination of cyclophosphamide/vinorelbine is used within the EpSSG for metastatic RMS. In North America, the COG does not currently regard maintenance therapy as the standard of care for metastatic RMS; however, COG study protocols include much longer absolute durations of therapy. There are considerations as to whether the concept of maintenance therapy or absolute duration of therapy is the more relevant metric for treatment.

In cases of metastatic disease with distant spread of disease from the primary tumor site, the value of localized treatment of the primary tumor is often overlooked. A European retrospective study demonstrated that aggressive localized treatment of the primary tumor (combined surgical resection and RT compared to either alone) led to improved outcomes in patients with metastatic RMS (70). Another retrospective study found that local treatment to all metastatic sites in stage IV RMS was associated with an improved progression-free survival (PFS) and overall survival (OS) at 5 years (71). These lines of evidence support the importance of strong localized therapy at both primary and metastatic sites. However, extended local therapy (RT or chemotherapy) is not always feasible in patients. Other directions that are currently being considered include targeting genetically quiescent cells with the administration of oral maintenance therapy (69) and efforts to design therapeutic agents specifically targeted toward the metastatic phenotype (72, 73).

### Relapsed RMS

Approximately one-third of pediatric RMS patients will experience progressive disease or relapse, with a median time to relapse/progression of 13 months from initial diagnosis (74). Based on multivariate statistical modeling, it is now possible to predict the chance of salvage following first relapse of localized RMS based on a number of factors associated with worse outcome: metastatic status, prior exposure to RT and chemotherapy, unfavorable size and site of the tumor, lymph node involvement, alveolar histology, and shorter time to relapse (75). In general, most relapsed RMS patients are treated with chemotherapy and local control (surgery and/or RT). Knowing the chance of salvage on a case-by-case basis is important for deciding treatment options for each patient. For instance, patients with a very low chance of cure will not respond effectively to salvage therapy, so these patients should be prioritized for enrollment in experimental trials (75). Meanwhile, patients who demonstrate relapse after low-risk disease may benefit from salvage chemotherapy, such as irinotecan/vincristine or alternating vincristine/doxorubicin/cyclophosphamide, and etoposide/ofosfamide (76, 77). Disappointingly, phase II trials for children with relapsed RMS have not demonstrated meaningful, single-agent activity of targeted inhibitors, such as a monoclonal antibody against IGF-1R (R1507) and a multi-kinase inhibitor, sorafenib (78, 79). Clearly, there is a need to understand why these therapeutic agents which show promise in the pre-clinical stage fail to translate into meaningful outcomes in patients, and to identify strategies targeting resistance mechanisms hindering their clinical efficacy. At any rate, novel therapeutic targets (Table 1) that are backed by supportive clinical evidence should also be explored as experimental options for patients with relapsed RMS.

**Table 1:** Current targeted therapies and immunotherapies targets under evaluation in preclinical and/or clinical development in North America and Europe for rhabdomyosarcoma.

**Molecular target**	**Drug**	**Phase**	**Clinicaltrialsregister.eu identifier (European)**	**Clinicaltrials.gov identifier (USA)**
BRD4	JQ1, OTX015	Preclinical		NA
CHD4	ED2-AD101 (SMARCA5/CHD4 dual inhibitor)	Preclinical		NA
HDAC	Entinostat, Vorinostat	Clinical (I/II)	2008-008513-19; 2018-000127-14	NCT02780804 (Entinostat
ALK	Critotinib	Clinical (II)	2011-001988-52	
PLK1	Volasertib	Preclinicial		NA
GSK3β	Tideglusib, LY2090314, 9-ING-41	Preclinical		NA
PI3K/mTOR	Omipalisib, Temsirolimus	Clinical (I/II)	2007-000371-42	NCT00106353; NCT01222715
MEK1	Cobimetinib	Clinical (I/II)	2014-004685-25	
FGFR	Erdafitinib	Clinical (II)		NCT03210714; NCT03155620
IGF-1R	R1507 (mAb)	Clinical (II)	2007-003940-30	NCT00642941
VEGF	Bevacizumab (mAb)	Clinical (II)	2013-003595-12	NCT01222715
Multi-RTKs	Regorafenib	Clinical (II)	2013-003579-36	NCT01900743
SMO	LDE225, Erismodegib, Vismodegib, Sonidegib	Clinical (II)	2010-019348-37	NCT01125800
NOTCH	RO4929097, MK0572, brontictuzumab (mAb), tarextumab (mAb)	Preclinical		NA
CDK4/6	Palbociclib, Ribociclib, Abemaciclib	Preclinical		NA
Wee1	AZD1775	Clinical (I/II)		NCT02095132
PARP	Olaparib, Iniparib, Veliparib	Clinical (II)		NCT03155620; NCT03233204
Bcl-2	Venetoclax, ABT-737	Preclinical		NA
XIAP	Smac mimetics (LCL161)	Preclinical		NA
**Immunotherapy**				
B7-H3	Enoblituzumab (mAb)	Clinical (I)		NCT02982941
HER2	Autologous HER2-specific CAR T cells	Clinical (I)		NCT00902044
EGFR	Autologogous EGFR-specific CAR T cells	Clinical (I)		NCT03618381
CTLA-4	Ipilimumab	Clinical (I/II)		NCT02304458; NCT01445379
PD-1/PD-L1	Nivolumab, Pembrolizumab, Atezolizumab	Clinical (I/II)	2014-004697-41; 2018-000127-14	NCT02304458

**Figure 2:**
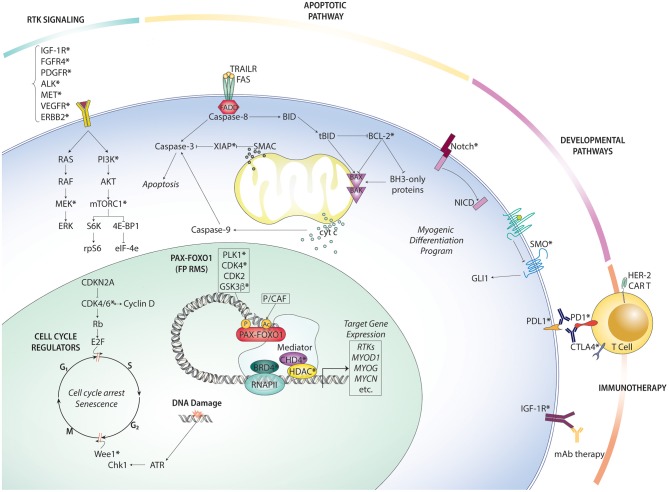
Overview of rhabdomyosarcoma targeted therapies organized by pathway. Therapeutically actionable targets (at least one existing small molecule inhibitor or antibody) are indicated with an asterisk (*).

Given the inherent limitations of therapeutic options available for metastatic and recurrent RMS, experimental trials should prioritize patients with metastatic or recurrent disease, including emerging targeted therapy and immunotherapy strategies. Below, we summarize the key preclinical and clinical findings on novel targeted therapy and immunotherapy options in RMS (Figure 2).

## Targeted Therapy

### PAX-FOXO1

#### Directly Targeting PAX-FOXO1 Fusion Protein

In FP RMS, the chimeric transcription factor, PAX-FOXO1 presents the most direct and promising target. Conceptually, it is more effective to target one upstream transcription factor than multiple downstream signaling cascades and hundreds of target genes. Moreover, because PAX-FOXO1 fusion protein is uniquely expressed in tumor cells but not in normal cells, it is an attractive target. Small molecule inhibitors against oncogenic fusion proteins have achieved remarkable clinical success in some human cancers, such as the targeting of BCR-ABL in leukemia and EML4-ALK1 in lung carcinoma (80, 81). Until recently, TFs were considered to be an “undruggable” class of proteins due to an absence of deep hydrophobic pockets, large protein-protein interaction interfaces, and nuclear localization (82). Today, the opinion on drugging transcription factors is beginning to shift [reviewed by (83)], as approaches to inhibit transcription factors have demonstrated some success in preclinical and clinical studies. Novel approaches to drug transcription factors are currently being investigated in other disease contexts, with the possibility of adapting these strategies for targeting PAX-FOXO1. One experimental approach is the use of nanoparticle carriers to deliver naked siRNA or antisense oligonucleotides (ASO) into tumor cells to silence specific genes. The advantage of this approach is that any gene can theoretically be targeted by simply knowing the complementary base pairing for the gene of interest. A recent publication demonstrated that liposome-protamine-siRNA (LRP) particles targeting PAX3-FOXO1 were efficiently delivered to ARMS cell lines and downregulated PAX3-FOXO1 and its target genes *in vitro*, leading to delayed tumor growth and inhibition of tumor initiation in ARMS xenograft models (84). However, safety concerns over off-target effects by the RNAi transcripts and the toxicity of delivery systems remain significant obstacles to translation of this approach into the clinic.

To date, there have not been significant efforts to design inhibitors which directly bind PAX-FOXO1. Yet, emerging strategies to directly drug transcription factors are currently being explored in other human cancers. These new approaches include modulation of the auto-inhibitory state of transcription factors, proteolysis targeting chimeras (PROTACs), use of cysteine reactive inhibitors, and targeting intrinsically disordered regions [reviewed in (83)]. Notably, PROTAC uses a bifunctional molecule simultaneously targeting the protein of interest and engaging an E3 ubiquitin ligase to promote proteasomal degradation of the target. So far, it has not been explored in RMS, but the documented efficacy in other studies support its consideration for targeting PAX-FOXO1 (85–87). The advantage of the PROTAC approach over traditional pharmacological inhibition is that a single molecule could be used for multiple rounds of proteasome-targeted degradation. The first step would be to identify ligands capable of binding PAX-FOXO1 with sufficient specificity and affinity. This is more feasible than other inhibitory approaches, since the ligand only needs to bind to a tractable surface, rather than a specific functional site which is much harder to target.

#### Coregulators of PAX-FOXO1

An alternative approach to disrupting PAX-FOXO1 activity is to target essential protein-protein interactions with co-regulators and chromatin-remodeling proteins required for oncogenic transcriptional activity. PAX-FOXO1 acts as a pioneering TF to establish looped super-enhancers, recruiting chromatin remodeling proteins, coactivators, and other TFs to aberrantly drive transcription at target genes. Only a small subset of TFs form the core regulatory circuit of TFs, which cancer cells are uniquely dependent on (88, 89). In FP ARMS, PAX-FOXO1 orchestrates the formation of super-enhancers, which drive the transcription of core regulatory TFs in a strong autoregulatory loop. A promising therapeutic approach is to disassemble the super-enhancer with small molecule inhibitors, thereby disrupting the oncogenic core regulatory circuit (90).

The selective disruption of super-enhancers by small molecule inhibitors can specifically suppress transcription at key oncogenic drivers (91). Gryder et al. was first to demonstrate a mechanistic link between the chromatin reader, BET bromodomain-containing protein (BRD4) and PAX3-FOXO1. The authors show that BRD4 small molecule inhibitor, JQ1 selectively disrupts the interaction between BRD4 and PAX3-FOXO1, leading to rapid degradation of the fusion gene and abrogation of transcriptional output (89). Another study reported that the antitumor activity of JQ1 is mediated by a decrease in angiogenic activity (92), which is consistent with the hypothesis that disruption of the super-enhancer ablates transcriptional output of gene targets, one of which is vascular endothelial growth factor (VEGF). Among the five structurally diverse BET bromodomain inhibitors tested in this study, OTX015 was reported to be most potent across a range of FP RMS cell lines, but its clinical efficacy has not been evaluated. A related therapeutic strategy targets chromatin helix DNA binding protein 4 (CHD4), an ATP-dependent chromatin remodeling protein which plays an integral role in the Nucleosome Remodeling Deacetylase (NuRD) complex. CHD4 is required for the recruitment of the transcriptional machinery, and its role in nucleosome eviction is required for transcription to proceed. (93) Upon knockdown of CHD4 *in vitro*, gene expression profiling showed that CHD4 activity is essential for the expression of a subset of PAX3-FOXO1 target genes, and that the observed effect was specific to FP RMS (93). Independently, another group found that CHD4 acts as a crucial coregulator of PAX3-FOXO1 (identified as a top candidate from a siRNA screen of 60 candidate interactors), suggesting the role of CHD4 as a therapeutic target in FP RMS (93). A first-in-class inhibitor (ED2-AD101) of SMARCA5/CHD4 was recently shown to suppress cell growth in acute myeloid leukemia (AML) cells, but no inhibitors specifically targeting CHD4 are currently available for clinical use (94).

Other studies have implicated that inhibition of another epigenetic regulator, histone deacetylase (HDAC) has antitumor effects in preclinical RMS models. A recent study designed a screen for epigenetic chemical probes to differentiate between super-enhancer driven transcription and constitutive transcription, revealing that the acetylation-axis is more important for the core regulatory TF circuit than the methylation-axis (90). Independent studies have reported the ability of HDAC inhibitors, entinostat, panobinostat, and vorinostat to delay tumor growth in xenograft models of RMS (95, 96). Several HDAC inhibitors are already approved for treatment of other cancers, but early clinical data show that HDAC inhibitors against solid tumors are far less effective than against hematological diseases, likely due to the pharmacokinetic differences in these two different tumor contexts (97). An ongoing clinical trial (NCT02780804) of the HDAC1/2/3 inhibitor entinostat in pediatric patients with advanced solid tumors is expected to shed new insight on HDAC inhibitors in RMS. Beyond disrupting transcriptional complexes to suppress the expression of key oncogenic genes, perturbation with HDAC inhibitors has also been shown to induce transcriptional chaos in cancer cells, driving cells into terminal differentiation or apoptotic cell death (98).

Taken together, these studies illustrate that FP RMS cells are differentially sensitive to the targeted disruption of super-enhancer complexes. Importantly, the knowledge that FP RMS cells are selectively dependent on epigenetic readers, writers, and erasers of the histone acetylation-axis can be exploited in the pharmacological disruption of these complexes (90). One important limitation to this approach is that epigenetic regulators also play a role in normal cellular transcriptional programs, meaning that there is a narrow therapeutic window and an increased risk of dose toxicity compared to an approach which directly targets the chimeric transcription factor.

#### Modulators of PAX-FOXO1 Activity and Stability

A third approach is to target regulatory post-translational networks regulating the activity and stability of PAX-FOXO1. Many of these post-translational modification sites (phosphorylation, acetylation, methylation) in the fusion protein have been identified through high throughput mass spectrometry experiments or *in vitro* enzymatic screens performed in wildtype FOXO1 TF (99). However, only some of these sites have been functionally validated. Of these, most are phosphorylation sites which are targeted by common kinases, and many of these kinase are druggable by known kinase inhibitors. In general, there are two approaches for targeting the regulatory networks of PAX-FOXO1; (1) targeting the regulatory kinases that influence protein stability, and (2) targeting the regulatory kinases required for activation of the fusion protein.

Several phosphorylation sites are known to influence protein stability of the fusion product. In one study, a two-armed screening approach of kinome siRNA and small molecules identified that the kinase PLK1 stabilizes the fusion protein by phosphorylating S503. Inhibition of PLK1 directly led to ubiquitination of the fusion protein, followed by rapid proteasomal degradation (100). This evidence is consistent with the known function of PLK1 in the cell cycle, which is to coordinate entry into mitosis at the G_2_/M checkpoint. The expression levels of PAX3-FOXO1 and PLK1 both peak in G_2_ in a cell-cycle dependent manner. Upregulation of PAX3-FOXO1 transcripts and its stabilization by PLK1 phosphorylation permit the cell to progress past the G_2_/M checkpoint (101). Another upstream enzyme, acetyltransferase KAT2B (P/CAF) is known to modulate fusion protein stability by acetylating residues K426 and K429 (102). Other phosphorylation sites are known to control the transcriptional activity of PAX3-FOXO1, including the residues S201 (phosphorylated by the kinase GSK3β) (103), S205/S209 (by CK2) (104), and S430 (by CDK4) (105). Notably, several GSK3β inhibitors significantly suppressed transcriptional activity of PAX3-FOXO1, leading to inhibition of cellular proliferation and induction of apoptosis in ARMS cell lines (106). Given the availability of kinase inhibitors that have been studied in other human cancers, further functional validation of post-translational modifications of PAX3-FOXO1 and characterization of their respective kinases is a promising therapeutic strategy.

### Receptor Tyrosine Kinases (RTK)/RAS/PI3K Axis

Receptor tyrosine kinases are a family of membrane-bound cell surface receptors which are aberrantly activated in many human malignancies. Constitutive activation of RTK signaling can reprogram numerous intracellular signaling pathways (metabolism, differentiation, apoptosis, growth) to promote tumor progression (Figure 2). In FP RMS, overexpressed PAX-FOXO1 targets include RTKs e.g., FGFR4, CXCR4, IGFR1, MET, and PDGFRA. In FN RMS, activating mutations in RTKs caused by molecular lesions can lead to hyperactive RTK signaling. Taken together, both FP and FN RMS could benefit from targeting RTK signaling. Generally speaking, the two strategies for targeting RTKs include small molecule kinase inhibitors and immunotherapy (monoclonal antibodies, CAR T). Several studies have shown the that RTK inhibitors can induce tumor regression in preclinical models (summarized in Table 1). Despite that many of these candidate targets are currently being evaluated in early phase I/II trials which recruit RMS patients, there has only been one clinical trial opened specifically for RMS patients. This trial (NCT03041701) is open to patients with relapsed or refractory RMS and its aim is to study the combination of the IFG-1R monoclonal antibody, ganitumab in combination with the SRC family kinase inhibitor, dasatinib. This treatment combination was based on preclinical evidence which reported that IGF-1R inhibition promotes a bypass resistance pathway through other kinases, such as the SRC family kinase YES (107) and ALK (108), suggesting that targeting multiple RTKs in combination is likely necessary to overcome resistance.

Despite the remarkable genetic and molecular differences between FP and FN RMS, the RTK/RAS/PI3K axis is commonly hijacked by both, suggesting that targeting this axis presents a more general therapeutic approach which could benefit a wide range of patients. A comprehensive genomic analysis of 147 RMS tumor samples by Shern et al. reported that the RTK/RAS/phosphoinositide 3-kinase (PI3K) pathway is altered in 93% of the tumors profiled, mostly dependent on the FGF and IGF receptor pathways (19). Fortunately, many of these pathway components (Table 1) can be targeted by clinically available therapeutics (19). The receptor tyrosine kinase, FGFR4 is frequently mutated and/or overactivated in RMS tumors, and recent work has implicated the role of FGF signaling in the evasion of apoptosis (109). Khan et al. identified potaninib from a panel of five tyrosine kinase inhibitors as a potent FGFR4 inhibitor that inhibits tumor growth in a RMS mouse model (110). The receptor tyrosine kinase, IGF2 is another potential target, given that either loss of imprinting or PAX-FOXO1-driven gene expression can induce the overexpression of IGF2 in rhabdomyosarcoma tumors (111). However, a recent clinical trial evaluating a monoclonal antibody against IGF-1R, R1507 in advanced sarcoma patients failed to achieve meaningful clinical responses to the therapy (79). Future studies should focus on elucidating potential resistance mechanisms to IGF-1R inhibition and identifying predictive biomarkers for IGF-1R inhibition sensitivity. A recent publication investigating a MEK inhibitor, trametinib in combination with IGF-1R inhibition showed a potent decrease in RMS cell viability and slowed tumor growth in xenograft models (112). Finally, a randomized phase II trial of bevacizumab (VEGF-A inhibitor) or temsirolimus (mTOR inhibitor) in combination with chemotherapy reported that the relapsed RMS patients who received temsirolimus achieved a better response (39). Although the RTK/RAS/PI3K axis is a common platform for therapeutic intervention in both FP and FN RMS, there is still a need to identify predictive biomarkers of response. Due to the extensive cross-talk across RTK signaling axes, combination therapies are likely needed to derive therapeutic benefit from this approach. Most of the data on targeting the RTK/RAS/PI3K axis in RMS comes from scattered preclinical reports, and none have demonstrated significant single agent activity in early phase clinical trials.

### Developmental Pathways

The knowledge that key developmental pathways, such as Hedgehog and Notch are commonly hijacked in a subset of RMS tumors can be exploited for therapeutic intervention. The Hedgehog (Hh) pathway is a highly conserved developmental pathway, which plays crucial roles in embryonic development, stem cell biology, and tissue homeostasis (113). In the canonical Hh pathway, repressive binding of Smoothened (Smo) to the transmembrane receptor Patched1 (PTCH1) maintains Hh signaling in an inactive state. Hh ligand binding to PTCH1 releases Smo, which becomes free to activate the Gli family of transcription factors (114). The link between Hh signaling and RMS was first described by Hahn et al. in a study of a *Patched* knockout mouse model that showed an ERMS phenotype. The relationship between Hh signaling dysregulation and RMS has subsequently been supported by several studies (115–118). Aberrant Hh signaling can be attributed to various germline mutations— loss of chromosomal region 9q22 containing *PTCH* in 33% of ERMS tumors (119, 120), loss of SUFU in 18% ERMS tumors (121), and/or genomic amplification of 12q13-15 containing the *GLI1* gene in a small subset of ARMS tumors (116). Several studies have demonstrated that targeting the Hh pathway can inhibit tumor growth and impair tumor initiation in xenografted RMS models (118, 122–124). So far, preclinical evaluation of Smo inhibitors has been difficult to interpret due to the heterogeneity of response in preclinical models, depending on the RMS cell line and Smo inhibitor assessed (125, 126). Recent FDA approval of the Smo inhibitors, vismodegib, and sonidegib for the treatment of advanced basal cell carcinoma (BCC) and entry of other Smo inhibitors into clinical trials for pediatric medulloblastoma raise the possibility of expanding these inhibitors into clinical trials for pediatric RMS (127). However, because only a subset of RMS tumors appear to be sensitive to Smo inhibitors (such as ERMS tumors with a germline *PTCH* mutation), more robust predictive biomarkers for this therapy need to be established (126). Concerns over the side effects of Smo inhibitors, such as premature closure of bone growth plates may limit their use to only skeletally mature patients (128).

The Notch pathway regulates cell fate determination and stem cell differentiation during tissue development and maintenance. A recent publication used a zebrafish transgenic model of ERMS to identify intracellular NOTCH1 (ICN1) as an important regulator of balancing self-renewal and differentiation in ERMS (129). This work highlights the mechanistic underpinnings of the NOTCH1/SNAI1 pathway in driving self-renewal and blocking MEF2C regulated myogenic differentiation in RMS, describing a rationale for targeting the NOTCH1/SNAI1/MEF2C axis in ERMS. Another study highlighted that downregulation of Notch3 is sufficient to induce RMS cells into a terminal myogenic differentiation program, suggesting Notch3 as another potential therapeutic target (130). One group reported that the oncogenic signaling circuit between the Notch and YAP pathways drives stemness and tumorigenesis in ERMS, suggesting a rationale for co-targeting Notch and YAP (131). Given that there are clinically available gamma-secretase/Notch signaling pathway inhibitors (RO4929097), Notch1 inhibitors (MK0752), and Notch1 monoclonal antibodies (brontictuzumab, tarextumab), these drugs should be expanded into clinical trials for pediatric RMS.

### Cell Cycle Regulators

In order to sustain chronic proliferation, cancer cells must meet the demanding needs imposed by energy metabolism and cellular division. The family of cyclin dependent kinases (CDK) which tightly control the cell cycle are frequently overactive in cancer cells, and have been extensively investigated as a molecular vulnerability in various human cancers. Several selective CDK4/6 inhibitors have already been approved for treatment in advanced stage breast cancer (132), and have been investigated in the context of RMS, as CDK4 is overexpressed in a subset of FP RMS tumors through the amplification of chromosomal region 12q13-q14 (133). CDK4 and its binding partner Cyclin D are required for progression through the G_1_/S checkpoint, and its overexpression allows cancer cells to adapt to the high proliferation rates needed to sustain tumorigenesis. One study showed that treatment of rhabdomyosarcoma cell lines and xenograft models with the clinically available CDK4/6 inhibitor, palbociclib is sufficient to inhibit proliferation by inducing cell cycle arrest at G_1_.

### DNA Damage Response (DDR) Pathway

The DNA damage response (DDR) pathway plays a critical role in normal cellular homeostasis and its dysregulation is closely linked to increased mutation rates which drive oncogenesis. The poly(ADP-ribose) polymerases (PARP) belong to a family of DNA damage sensors which target the poly(ADP-ribose) polymerase by binding to single strand DNA breaks, recruiting other components of the homologous recombination (HR) repair machinery (134). PARP inhibitors (PARPi) are a well-established class of compounds capable of abrogating single strand break repair, which are converted into double strand breaks, subsequently leading deficient DNA repair and cell death (135). The understanding that inhibition of the DDR can be exploited in cancer cells to sensitize them to DNA lesions induced by chemotherapy or RT has been well-established in other cancers. However, the role of PARP inhibitors in RMS has not been extensively studied. A recent study showed that PARP inhibitors can sensitize RMS cell lines to ionizing radiation (IR), resulting in more potent cytotoxic effects compared to either modality alone (136). A related study reported that pretreatment with three PARP1 inhibitors (olaparib, iniparib, veliparib) was able to sensitize soft tissue sarcoma cells to radiation by inducing cell cycle arrest at the G_2_/M checkpoint (137). Taken together, these data point to the effective approach of combining PARP inhibitors with radiotherapy, sensitizing cancer cells to the ionizing radiation and tolerating lower doses of radiation. A recent preclinical study reported that the combination of olaparib and temozolomide (DNA-damaging agent) is a potent therapy for elimination of tumor cells in a human xenografted tumor zebrafish model of RMS. Both ERMS and ARMS were sensitive to combination treatment, suggesting the broad therapeutic potential of PARP inhibition in RMS (138). While combination therapy of olaparib and temozolomide is currently being investigated in phase II trials for Ewing's sarcoma, there are no open trials for this combination treatment in rhabdomyosarcoma. The strong preclinical evidence for the combination therapy of olaparib and temozolomide (138) warrants further investigation in clinical studies focused on pediatric RMS.

The Wee1 kinase arrests the cell cycle at the G_2_/M checkpoint for necessary DNA repair before entry into mitosis. Because cancer cells are more reliant on the G_2_/M checkpoint for DNA repair than normal cells due to G_1_/S DNA repair deficiencies, Wee1 inhibition can halt progression through the G_2_/M checkpoint and selectively induce apoptosis in cancer cells. One preclinical study reported that Wee1 kinase inhibitor AZD1775 possessed single-agent activity and synergized with conventional cytotoxic therapy (139). Wee1 inhibition against a background of cytotoxic drug-induced DNA damage results in mitotic catastrophe in tumor cells. A recent study used an integrated transcriptomic, epigenomic, and proteomic approach based on orthotopic patient-derived xenografts to validate and prioritize specific molecular vulnerabilities for high-risk RMS. The authors identified Wee1 kinase to be the most significant target for high-risk RMS, which led them to propose that patients with high-risk and recurrent RMS may benefit from combination therapy that includes AZD1775, irinotecan, and vincristine (140). Based on comprehensive preclinical testing data, patients with high-risk pediatric RMS were included in a phase I/II clinical trial (NCT02095132) of AZD1775 in combination with the chemotherapy agent irinotecan. A recent consensus article by American and European RMS leaders argued for the prioritization of the WEE1 inhibitor AZD1775 in combination with vincristine/irinotecan for the next clinical trial for patients with initially metastatic or recurrent RMS (141).

### Apoptosis Pathway

Direct modulation of apoptotic machinery has been exploited therapeutically in many human cancers, as most cancer cells are more sensitive to apoptotic induction than normal cells (142, 143). Because the Bcl-2 family of antiapoptotic proteins is required for cancer cell survival, inhibiting its function is one potential therapeutic approach. The development of BH3 mimetics, small molecule inhibitors which mimic the function BH3-only proteins by antagonizing the pro-survival function of anti-apoptotic Bcl-2 family, has recently gained traction as a therapeutic intervention in a number of human cancers. The Bcl-2 inhibitor, venetoclax is currently under evaluation in a trial for pediatric neuroblastoma and hematological malignancies (NCT03236857). Preuss et al. showed that ABT-737 (BH3 mimetic) and AZD8055 (mTOR inhibitor) cooperate synergistically to induce the mitochondrial apoptotic pathway in ERMS and ARMS cell lines (144). Another study showed that venetolax sensitized RMS cells to JNJ, an HDAC inhibitor (145). Taken together, these findings suggest that BH3 mimetics synergize with other targeted therapies by priming cancer cells to be sensitive to apoptotic induction.

A related strategy is the inhibition of X-linked Inhibitor of Apoptosis Proteins (XIAP), a family of proteins which block apoptosis by directly binding and inhibiting caspases, and which are frequently overexpressed in cancer cells. Smac mimetics are a class of molecules designed to mimic the endogenous antagonist of XIAPs, second mitochondrial activator of caspases (Smac). Upon activation of the mitochondrial apoptotic pathway, Smac is released into the cytosol, where it binds and neutralizes XIAPs, thereby allowing the caspase cascade to proceed. One study reported that Smac mimetics sensitized two RMS cell lines toward natural killer (NK) cell-mediated killing in an apoptotic-dependent manner (146). Currently, several Smac mimetics are being evaluated in early clinical trials for other human cancers but there are no open trials for RMS (147).

## Immunotherapy

### Targeting PAX-FOXO1 as a Tumor Antigen

The recurrent 2;13 and 1;13 translocations in FP RMS encode for the PAX3-FOXO1 and PAX7-FOXO1 chimeric transcription factors, which are uniquely expressed in malignant cells but not in normal cells. This raises the possibility that the PAX-FOXO1 chimeric proteins can be leveraged as novel tumor-associated antigens in immunotherapy. The unique translocation breakpoint region may be processed, displayed on the tumor cell surface by major histocompatibility complex Class I (MHC-I) molecules, and targeted for killing by cytotoxic T cell lymphocytes (CTL) (148, 149). While data from preclinical mouse studies highlighted the vaccine-based approach as a promising strategy (149), a pilot clinical trial that generated vaccines by pulsing immature dendritic cells from breakpoint region peptides failed to improve patient outcomes (150). A separate study generated a human CTL line capable of lysing HLA-B7 rhabdomyosarcoma tumor cells (151). However, due to the limitation that this vaccine would only be applicable to the minority of the population who express the HLA-B7 allele, its clinical potential is limited. Attempts to generate vaccines targeted against other class I molecules including HLA-A1, HLA-A2, HLA-A3 are unlikely to be successful, as predicted by MHC-peptide binding algorithims (152). A pilot trial of consolidative immunotherapy (integration of immunotherapy into a multi-modal chemotherapeutic regimen), which administered vaccines of dendritic cells pulsed with breakpoint peptides reported positive outcomes in patients with high-risk pediatric ARMS, highlighting that vaccine-based approaches targeting the fusion protein could still be a valuable strategy. However, there is still room for improvement in refining this approach, such as using alternative approaches for generating more potent dendritic cells and identification of immunogenic peptides (153). New models that predict the immunogenicity of MHC-binding peptides from tumor transcriptomes can be leveraged to identify novel immunogenic peptides (154).

### Monoclonal Antibodies

In the last two decades, monoclonal antibodies (mAbs) have become standard-of-care treatment of several human malignancies, but its role in RMS is not well established. Monocolonal antibodies can directly target cancer cells through a number of mechanisms, including inhibition of oncogenic signaling pathways, delivery of cytotoxic moieties to malignant cells, or induction of antibody-dependent cellular toxicity (155). One strategy has been the development of an Fc-enhanced anti-B7-H3 monoclonal antibody to target the B7-H3 protein upregulated on the surfaces of many pediatric solid tumor cells (156). Based on support from preclinical testing, a Phase I study was opened to evaluate enoblituzumab, an Fc-enhanced, humanized IgG1 monoclonal antibody specific for B7-H3 and engineered with an Fc domain with increased affinity for the activating receptor CD16A, thereby enhancing antibody dependent cellular cytotoxicity (ADCC) (157). Compared to classical mAb therapies which are less effective due to the existence of natural polymorphisms of FcγR, the strategy of fine tuning the Fc domain to optimize effector cell function should be considered in mAb-based approaches for RMS.

### CAR T-Cells

Early phase clinical studies of CAR T cell therapy for patients with pediatric solid tumors has demonstrated that while it can be safely administered, antitumor activity is limited (158). Targeting the human epidermal growth factor receptor 2 (HER2) expressed on tumor cells with the anti-HER2 antibody trastuzumab is an established therapy for the treatment of HER2-positive breast cancers (159, 160). Given that a subset of sarcomas express HER2 on tumor cell surfaces, targeting this receptor by immunotherapy is one potential strategy. Because HER2 expression levels are too low in sarcoma cells for a monoclonal antibody-based approach to be therapeutically actionable, HER2-positive sarcoma patients may be more sensitive to HER2-directed CAR T cell therapy (161). Following preclinical evaluation of a HER2-specific CAR containing a CD28.ζ signaling domain, Navai et al. (162) conducted a small phase I study (NCT00902044) evaluating the efficacy of HER2-targeted CAR T-cell therapy in combination with lymphodepletion chemotherapy in patients with advanced HER2-positive sarcoma. One pediatric RMS patient treated achieved a complete response for 12 months, but relapsed later (163). Another recent preclinical study of CAR T cells targeting B7-H3 (an immune checkpoint antigen) in xenograft models of various pediatric solid tumors, including RMS demonstrated that they could induce tumor regression in xenograft models (164). Other potential cell surface immune targets (FGFR4, SLC19A1, ACVR2A, EPHB4) were identified by Khan et al., in a study which used gene expression datasets to rank potential immune targets by their differential expression between 12 pediatric cancer tissues and normal tissue (165). Such gene expression approaches can be a useful strategy to generate a list of possible immune targets, but validation that these targets are actually expressed at the protein level on tumor cells (and not expressed on normal cells) is required before they are considered for CAR T therapy.

Compared to the success of CAR T cell therapy for treating B-cell-derived malignancies, the clinical efficacy of CAR T cell therapy to pediatric solid tumors has so far been limited. As reviewed by DeRenzo et al., treatment of solid pediatric tumors presents a unique set of challenges that must be carefully taken into consideration. Challenges of designing CAR T cell therapy for solid tumor malignancies include: heterogeneous antigen expression, limited migration of T cells to tumor sites, and an immunosuppressive, hostile microenvironment (158). In order to advance the field of CAR T cell therapy in pediatric solid tumors, there is a need for further optimization of CAR T cells at the preclinical stage, identification of immunogenic targets, and a technique to non-invasively monitor CAR T activity in patients in the clinical trial stage (158).

### Immune Checkpoint Blockade

Cytotoxic T-lymphocyte-associated protein 4 (CTLA-4) and programmed cell death protein 1 (PD-1) belong to a class of inhibitory receptors, which are negative regulators of T-cell immune function. In a tumor context, cancer cells have evolved mechanisms to co-opt this system, enabling cancer cells to evade immune surveillance. For example, when PD-1 receptor on T cells is engaged by its native ligand, PD-L1, T cell effector function is inhibited. Thus, tumor cells have evolved to express PD-L1 on their surfaces to deactivate T cell effector function, enabling them to evade destruction by the immune system. Release of the negative regulators at these checkpoints with checkpoint blockade therapy can induce a latent anti-tumor immune response (166). Given the clinical success of immune checkpoint inhibitors in metastatic melanoma and metastatic squamous non-small cell lung cancer, early-phase clinical trials are currently investigating their clinical efficacy in pediatric solid tumors (167–169).

Ipilimumab is a first-in-class anti-CTLA-4 immune checkpoint inhibitor approved for treatment of metastatic melanoma and was recently evaluated in a phase I clinical trial for the treatment of pediatric advanced solid tumors. Even though ipilimumab is safely tolerated in these patients, its efficacy as a monotherapy is limited. The authors of this study recommend future investigation of anti-CTLA-4 therapy in combination with other checkpoint inhibitors and/or immune-modifying agents (170). Targeting the PD-1/PD-L1 axis by mAb therapy (nivolumab, pembrolizumab) has a similar mechanism to CTLA-4 therapy, in which brakes are released on the anti-tumor activity of T cells. A pilot phase I trial (NCT01445379) of ipilimumab in children with advanced refractory solid tumors showed that no objective tumor regressions were achieved (170). Combination therapy of both CTLA-4 and PD-L1 inhibitors have demonstrated clinical efficacy in advanced melanoma, suggesting its consideration in pediatric cancers (171, 172). Initial data from an ongoing phase I/II trial (NCT02304458) evaluating nivolumab with/without ipilimumab in children with recurrent or refractory solid tumors or sarcomas showed that single-agent nivolumab has no activity, but in combination with ipilimumab demonstrated efficacy in certain sarcoma subtypes (173).

It is unlikely that immune checkpoint blockade in pediatric patients will achieve the same levels of response seen in adult patients. In adult cancers, a high mutational burden (more neoantigens) is correlated with a strong response to immune checkpoint inhibitors. Pediatric cancers are characterized by a low mutational burden, but it may be interesting to study whether RMS patients with higher mutational burdens (ERMS subtype) are more responsive to immune checkpoint therapy. Moreover, children do not yet have a fully developed immune system, which is required for optimal response to immunotherapy. Finally, the highly immunosuppressive microenvironment of pediatric sarcomas due to the presence of regulatory T cells and myeloid-derived suppressor cells limits the efficacy of immunotherapy.

## Future Directions—Personalized Therapy and Overcoming Drug Resistance

In an ideal world with unlimited financial resources and time, drug development efforts would be focused on developing pediatric cancer-specific drugs, such as a direct inhibitor of the PAX-FOXO1 fusion protein uniquely expressed in FP RMS. In reality, few drugs have been developed specifically for childhood cancers due to a small market for a rare childhood diseases, and the ability to directly target PAX-FOXO1 does not appear to be within reach for some time. For now, most clinical trials opened for RMS exploit known drugs targeting common pathways which are dysregulated in other human cancers (Figure 2). Careful review of how targeted therapies have been successful in clinical trials for other human malignancies [e.g., immune checkpoint blockade in metastatic melanoma (168, 169, 172, 174, 175)] and systematic analysis of clinical trials of related families of childhood cancers [e.g., PARP inhibition in Ewing's sarcoma (138)] provide valuable insight into translating these therapies into a RMS tumor context.

In FP RMS, one strategy to target PAX-FOXO1 has been the selective disruption co-regulatory and post-translational networks of PAX-FOXO1 with clinically approved inhibitors. While such approaches have shown encouraging responses in preclinical studies, these targets have normal physiological functions unrelated to the fusion protein, so careful consideration must be given to off-target effects. On the other hand, FN RMS (lacking the chimeric transcription factor) harbors recurrent genetic alterations (*RAS, FGFR4, IGFR1, CDK4, PI3KCA*, etc.), conferring these tumors molecular dependencies which can be targeted by clinically available drugs (19).

Based on data from early clinical trials enrolling pediatric rhabdomyosarcoma patients, single-agent therapies do not appear to achieve durable responses. As with most targeted therapies, almost all cancers treated with a single-agent therapy will eventually acquire resistance and reduced sensitivity to subsequent lines of treatment. Of equal importance to identifying novel therapeutic targets, is the unequivocal need to better understand how RMS tumors develop resistance to these therapies. Given the recent surge of research completed to characterize the genomic, epigenomic, transcriptomic, and proteomic landscape of rhabdomyosarcoma (117, 140), we now have a comprehensive list of therapeutic vulnerabilities, which are currently under preclinical and clinical evaluation. However, narrowly focusing on identifying these targets is inadequate, and a commensurate amount of effort ought to be given to studying mechanisms of resistance to targeted therapy. For patients diagnosed with metastatic rhabdomyosarcoma, insufficient local therapy options and incomplete eradication of occult microscopic residual disease are the most common causes of treatment failure (176). These new targeted therapies and immunotherapies hold promise for patients with metastatic or recurrent RMS, but only insofar as we concurrently advance our understanding of how to overcome inevitable drug resistance. While the identification of novel therapeutic vulnerabilities for RMS is gaining significant traction, it is equally important for clinicians to remain one step ahead by being able to anticipate resistance mechanisms and to identify strategies to overcome resistance accordingly.

Even the most successful targeted therapies that have been approved for the treatment of human cancers fail to completely eliminate residual disease in patients, leading to eventual relapse despite an initial response. A bulk solid tumor, such as rhabdomyosarcoma is composed of a heterogeneous population of cells which evolves to be more genetically unstable and complex as selective pressure is applied during drug treatment (177). Pediatric cancers are characterized by dynamic chromosomal instability, which can result in loss of chromosomal segments or copy-number alterations, contributing to the genetic heterogeneity of the tumor mass. A small subpopulation of drug-resistant tumor cells (harboring a genetic alteration conferring a survival advantage) present at initial treatment may persist and expand, resulting in eventual failure to eliminate residual tumor mass. Generally speaking, the two known strategies for overcoming drug resistance are intermittent dosing schedules and combination therapies. Intermittent dosing relies on the principle that periods of interspersed drug-withdrawal between drug-treatments can restore drug sensitivity by allowing drug-sensitive subpopulations to repopulate the tumor mass. Mathematical modeling can be used to predict the optimal dosing schedules which can sustain drug sensitivity, as demonstrated by a study which used an algorithm to design a tyrosine kinase inhibitor (TKI) dosing schedule for treatment of non-small cell lung cancers (178). These modeling platforms should be integrated into the design of future clinical trials for TKIs in RMS, where TKIs such as IGF-1R have demonstrated limited efficacy so far in early phase clinical trials (79, 179). A second approach is to anticipate which pre-existing subclonal populations will be drug-resistant, identify molecular vulnerabilities for these drug-resistant subclones, and design combination therapies focused on eradicating the maximal percentage of the heterogeneous tumor mass during first-line therapy. As an example, one study in non-small lung cell cancers showed that drug-resistant tumor cells with acquired resistance conferred by the EGFR^T790M^ mutation could be re-sensitized to EGFR TKIs by co-targeting them with navitoclax, an inhibitor of BCL-2 and BCL-XL (180). With the expansion of CRISPR-Cas9-based gene editing systems, we now have the ability to conduct unbiased genome-wide screens for therapeutic vulnerabilities in RMS to identify synthetic lethal combinations (181, 182).

Initial efforts to bring immunotherapies designed for adults into rhabdomyosarcoma pediatric trials has been met with limited success. These immunotherapies fail to translate because pediatric solid tumors are characterized by a lower mutational burden and a non-inflammatory tumor microenvironment (defined by very few infiltrating T cells and low levels of chemokines/cytokines) (183). Although further study is needed to elucidate the landscape of the RMS tumor microenvironment, pediatric solid tumors are generally regarded as less “inflamed” than adult tumors (184). As recently reviewed here (183), patients with “hot” tumor microenvironments (immune infiltrated) respond better to immune checkpoint blockade, whereas patients with “cold” tumor microenvironments (immune non-infiltrated) are better suited for adoptive T cell therapy. Further characterization of the tumor microenvironment of rhabdomyosarcoma would help guide the choice of an immunotherapeutic approach.

As whole-genome and transcriptome sequencing of rhabdomyosarcoma tumors has revealed, the genomic diversity of this disease requires a personalized (genotype-guided) approach to therapy. The presence or absence of the PAX-FOXO1 fusion gene reflects the vast genetic and molecular differences between FP and FN tumors, and this prognostic marker should guide the design of therapies specific to each subclass (37). Multi-region sampling of the tumor, single-cell sequencing, autopsy analysis, and longitudinal analysis of liquid biopsy samples can be used to reconstruct a holistic picture of tumor evolution in response to drug treatment, which can guide clinical decisions to counteract potential drug resistance (177). The gap between the observed efficacy of targeted therapies in preclinical models of RMS and the marginal improvements in patient outcomes observed in clinical trials has forced a reconsideration of our approach. Only when the mechanisms of drug resistance are understood will these new treatments be effective for children with metastatic or recurrent RMS, for which intensive chemotherapeutic regimens have already been exhausted.

## Conclusion

Our current understanding of the genomic and molecular landscape of rhabdomyosarcoma has equipped us with a valuable list of potential targets for targeted therapy and immunotherapy based approaches. Effective clinical translation of these agents remains an ongoing challenge, underscoring the need to elucidate why tumors eventually acquire resistance to targeted therapy. Given the small number of patients available for enrollment in clinical trials, it is necessary to prioritize which therapeutic targets and combination therapies will have the most clinical benefit for the greatest number of patients. The marked heterogeneity across RMS subtypes (and specifically within the ERMS subtype) means more personalized, genotype-guided approaches are needed to inform treatment decisions. As such, there is a need to identify reliable and objective biomarkers to determine the most effective therapy for each patient. With the inadequate outcomes observed in early RMS clinical trials of targeted therapies and immunotherapy, the conversation must shift toward how we can close the gap between the preclinical and clinical efficacy of these therapies.

## Author Contributions

CC, HD, MS, and AH contributed to the conception and design of the review. CC wrote the first draft of the manuscript. All authors contributed to manuscript revision, read, and approved the submitted version.

### Conflict of Interest

The authors declare that the research was conducted in the absence of any commercial or financial relationships that could be construed as a potential conflict of interest.
